# Pressurized-Liquid Extraction as an Efficient Method for Valorization of *Thymus serpyllum* Herbal Dust towards Sustainable Production of Antioxidants

**DOI:** 10.3390/molecules26092548

**Published:** 2021-04-27

**Authors:** Živan Mrkonjić, Dušan Rakić, Muammer Kaplan, Nemanja Teslić, Zoran Zeković, Branimir Pavlić

**Affiliations:** 1 Faculty of Technology, University of Novi Sad, Bulevar cara Lazara 1, 21000 Novi Sad, Serbia; zivan_mrkonjic@hotmail.com (Ž.M.); drakic@tf.uns.ac.rs (D.R.); zzekovic@tf.uns.ac.rs (Z.Z.); 2 TUBITAK Marmara Research Centre, Institute of Chemical Technology, P.O. Box 21, Gebze 41470, Kocaeli, Turkey; muammer.kaplan@tubitak.gov.tr; 3 Institute of Food Technology, University of Novi Sad, Bulevar cara Lazara 1, 21000 Novi Sad, Serbia; nemanja.teslic@fins.uns.ac.rs

**Keywords:** *Thymus serpyllum* L., pressurized-liquid extraction, polyphenols, antioxidant activity, multi-response optimization

## Abstract

The aim of this study was to valorize *Thymus serpyllum* herbal dust, a particular fraction distinguished as an industrial waste from filter-tea production. Pressurized liquid extraction (PLE) was used with the aim of overcoming certain obstacles of conventional extraction techniques in terms of shortening extraction time, reducing solvent consumption and energy costs, using “green” solvents and obtaining high yield and quality products. In order to optimize PLE of *T. serpyllum* herbal dust, the preliminary screening of the independent variables in order to define the most influential parameters and their domain was done first. After the screening, the optimization study using the face-centered central composite experimental design (CCD) with response surface methodology (RSM) was implemented. Additionally, taking into account the high awareness of the positive influence of antioxidants on the human health and associating it with high content of polyphenolic compounds in various members of Lamiaceae family, PLE has proven to be a great approach for antioxidants recovery from *T. serpyllum* herbal dust.

## 1. Introduction

*Thymus serpyllum* L., also known as a wild thyme, is an aromatic herb from the Lamiaceae family that is considered a valuable source for many different formulations in the pharmaceutical, food, cosmetic and chemical industry [1]. The main reason for this its long list of pharmacological properties, such as antiseptic, antibacterial, anthelmintic, diaphoretic, spasmolytic, expectorant, antitussive, analgesic, carminative and diuretic properties [2,3]. *T. serpyllum* has been studied widely for its antioxidant activity, which is directly correlated with a high content of polyphenolic compounds [4,5,6].

Due to the availability of large quantities of by-products of various industries, such as food, textile, pharmaceutical industry or agriculture, as well as their negative impact on the environment, in the recent years, emphasis has been placed on their reutilization. The recovery of polyphenolic compounds from by-products represents a great challenge, as well as opportunity for its commercial usage and utilization [7]. Considering the mean particle size of the herbal dust with less than 0.315 mm, this material cannot be further used for filter-tea production, and it is usually discarded from the factory as a by-product [8]. Although it is assumed that by-products from filter-tea industry possess fewer valuable bioactive compounds compared to final products of aforementioned industries, this material still possesses a significant amount of them, which has been confirmed through several studies [8,9,10].

Extraction techniques represent an excellent way for valorization of the *T. serpyllum* plant material [5,11,12,13]. In order to overcome certain obstacles of conventional extraction techniques (maceration [14], heat-assisted extraction [5], percolation [15] and Soxhlet extraction [12]) in terms of shortening extraction time, reducing energy costs, reducing operating temperature to preserve thermolabile bioactive substances, using “green” solvents and obtaining extracts with maximized polyphenols yield and bioactivity, pressurized liquid extraction (PLE) was used in this study. PLE represents extraction procedure that uses organic solvents at high pressures and temperatures above the boiling point [16]. The most recommended solvent for PLE is water and a mixture of water and ethanol, thanks to their properties in terms of an increased selectivity and reduced negative impact on the environment. The solubility of phenolic compounds is enhanced by ethanol, while water enhances desorption from the sample [17]. Increased temperature leads to a decrease in the viscosity of the solvent, which increases the ability of the solvent to access plant cells easily. High temperature also accelerates diffusion rate of target compounds to the matrix surface, ensuring fast and efficient extractions. In comparison with other extraction techniques, PLE has advantages in terms of time saving, automation, selective and efficient extraction utilizing low solvent volumes. Additionally, PLE has an advantage over some extraction techniques like ultrasound- and microwave-assisted extractions in terms of no requirement for additional filtration step of crude extract, which makes the down-stream of the process much easier [18]. PLE has so far proved to be a very good approach for isolation of polyphenols from Lamiaceae species, such as mint (*Mentha piperita* L.) [19], sage (*Salvia officinalis* L.) [20], rosemary (*Rosmarinus officinalis* L.) [21], thyme (*Thymus vulgaris* L.) [22] and wild thyme (*T. serpyllum* L.) [12].

In order to optimize the PLE of *T. serpyllum* herbal dust, screening experiments were needed to be done in the initial phase. Through the evaluation of PLE parameters that influenced on the target responses (total extraction yield (Y), total phenols yield (TP) and antioxidant activity determined towards DPPH radicals), the most influential PLE parameters were selected. After the screening, the main experiments were performed according to the multi-response optimization of polyphenols recovery from *T. serpyllum* using desirability function with Y, TP and antioxidant activity determined towards DPPH, FRAP (ferric ion reducing antioxidant power) and ABTS assays as target responses. Finally, the validation of the optimization process was done and the optimized extract and the extract obtained at the central point were subjected to HPLC-MS/MS analysis in order to determine their qualitative polyphenols profile.

## 2. Results and Discussion

### 2.1. Preliminary Experiment

PLE performances can be affected by numerous factors, such as temperature, extraction time, type of solvent and its concentration, cell flush volume and number of cycles [23]. Strategy for designing the extraction process in the initial phase of this study was to reduce the number of experiments and to determine the most influential parameters, as well as their domain for the further study. In order to accomplish that, the preliminary screening of the independent variables using 2^5-1^ fractional factorial design was done. The experimentally observed values of responses Y, TP and DPPH, using independent variables the temperature, extraction time, ethanol concentration, cell flush volume and number of cycles, are presented in Table 1.

**Figure 1:**
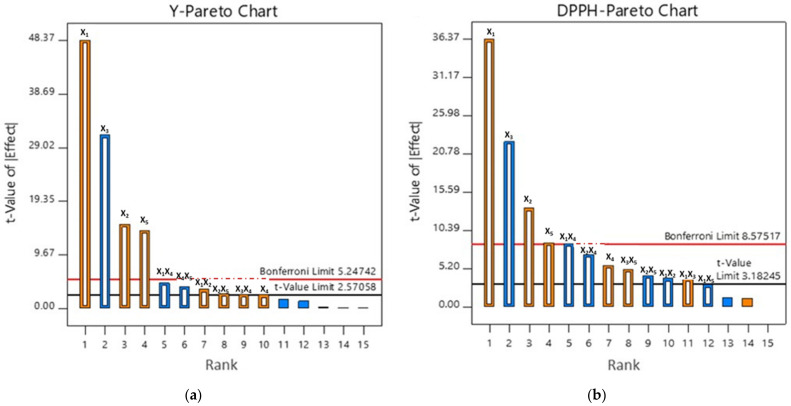
Pareto chart exhibiting effects of temperature (X_1_), extraction time (X_2_), ethanol concentration (X_3_), cell flush volume (X_4_) and number of cycles (X_5_) on (**a**) Y and (**b**) DPPH.

In order to present the effect of PLE variables on Y, TP and DPPH, the Pareto chart was used (Figure 1 and Figure S1).

Based on the t-values obtained for Y and DPPH, it could be concluded that the strongest impact on targeted responses was temperature, followed by concentration of ethanol and extraction time. According to t-values, number of cycles was also marked as significant, but it was still not taken into account in the response surface methodology (RSM) study, due to the fact that the differences in Y, TP, as well as antioxidant activity of the obtained extracts were negligible, with the substantially higher solvent consumption.

Correlation between increasing Y, TP and antioxidant activity of PLE extracts and temperature increase can be seen in Figures S2–S4, respectively. The same trend was reported by Hossain et al. [24], whose investigation was based on optimization of PLE of sage (*Salvia officinalis* L.), basil (*Ocimum basilicum* L.) and thyme (*Thymus vulgaris* L.), as well as by Zeković et al. [25], who optimized PLE of antioxidants from *Coriandrum sativum* seeds. Elevated temperature in combination with high pressure (1500 psi) and short exposure time prevents the degradation of thermolabile compounds, which is why PLE represents one of the most desirable extraction techniques [26]. Prolonged extraction time could also provide the higher Y, TP and antioxidant activity, but in combination with high temperature it could also accelerate the degradation of biologically active compounds [27]. For that reason, it is necessary to set upper limit values for temperature and extraction time taking into account the techno-economical aspect and rationalization of the PLE of *T. serpyllum* extracts. The negative influence of ethanol concentration on Y (Figure S2) was in agreement with finding of Miron et al. [4], who reported that target compounds from *T. serpyllum* could be efficiently extracted with 25% of ethanol or with pure water.

Through the evaluation of PLE parameters influence on the target responses, three out of the five of the most influential and the most appropriate PLE parameters were selected for the further RSM study of this research. A similar approach was implemented by Vakula et al. [28], who optimized ultrasound-assisted extraction (UAE) of vacuum-dried cornelian cherry using also the 2^5-1^ fractional factorial design for the screening of main UAE factors. After screening they determined the influence of process parameters and used the face-centered experimental design for the optimization of the UAE process. The positive influence of linear terms of temperature and extraction time, and negative one of concentration of ethanol on Y (Figure S2), TP (Figure S3) and DPPH (Figure S4) led us to the formation of the experimental domain for RSM study, that was precisely chosen as follows: temperature (130, 150 and 170 °C), extraction time (10, 20 and 30 min) and ethanol concentration (30, 45 and 60%).

### 2.2. RSM Study

Based on the results of the preliminary screening, a set of experiments of RSM study was designed with face-centered central composite design (CCD) with three levels of the previously chosen PLE parameters, while the cell flush volume and number of cycles were fixed at 50%and 1, respectively. In comparison with preliminary experiments, in the RSM study, apart from Y, TP and DPPH, antioxidant activity obtained by FRAP and ABTS assays were also used as responses, which experimentally observed values were presented in Table 2.

In order to check the adequacy and significance of the applied models, analysis of variance (ANOVA) was used (Table 3). In accordance with high values of coefficient of determination (*R*^2^) obtained for Y, TP, DPPH, FRAP and ABTS (0.992, 0.900, 0.845, 0.849 and 0.829, respectively), it could be suggested that there is a good fit between experimentally observed values and the values from applied quadratic model. In order to describe the dispersion degree of the data for investigated responses, the coefficient of variance (CV) was used. The values of CV for Y, TP, DPPH, FRAP and ABTS were 1.80, 5.47, 2.38, 4.51 and 5.47%, respectively, whose relatively low values indicated good fitness of the applied model. According to non-significant lack of fit (*p* > 0.05), the applied model indicated that it adequately describes the system, except for the ferric ion reducing antioxidant power (FRAP) of extracts (Table 3). A significant lack of fit in case of FRAP suggests that disagreement between the model and experimental data might occur and optimization should be confirmed by verification study. However, according to high value of *R*^2^ (0.849) and low value of CV (4.51%), as well as significant regression for the model (*p* < 0.05) (Table 3), it could be concluded that applied quadratic model represented good approximation of experimental results for FRAP assay, which further leads to the conclusion that RSM could be used for the optimization of the PLE process.

To our best knowledge, PLE of polyphenols from *T. serpyllum* using RSM has not been done before. Hossain et al. [24] did similar research, but with other plants from the Lamiaceae family. Furthermore, they applied CCD in order to investigate the effects of solvent concentration and extraction temperature on the TP and FRAP and to optimize PLE of polyphenols from sage (*S. officinalis* L.), basil (*O. basilicum* L.) and thyme (*T. vulgaris* L.). As a result, they concluded a very good agreement between the validated predicted model and actual experimental values [24].

In addition, ANOVA was used for calculation of *p* values of linear, interaction and quadratic terms for regression coefficients from the second-order polynomial model (Table S1). According to significance of linear, cross product and quadratic terms on Y, TP, DPPH, FRAP and ABTS, the reduced predictive model equations with neglected insignificant coefficients were presented in Table 4.

### 2.3. Total Extraction Yield (Y) and Total Phenols Yield (TP)

The extraction of polyphenols from *T. serpyllum* was already done by Jovanović et al. [5], who optimized three different extraction techniques by varying the particle size of the raw material, solid-to-solvent ratio, extraction time and type of solvent. The measured TP values obtained using maceration, heat-assisted extraction (HAE) and UAE were 26.6 mg GAE/L, 29.8 mg GAE/L and 32.7 mg GAE/L, respectively [5]. In comparison with other conventional and non-conventional extraction techniques, PLE has been proven to be a very efficient approach for extraction of polyphenolic compounds [12]. Y in the *T. serpyllum* extracts varied between 16.17 and 32.54%, while TP varied between 4.0400 and 6.6454 g GAE/100 g, depending on the different set of PLE conditions (Table 2). The highest Y was obtained at run 6, at 170 °C for 30 min using 30% of ethanol, while the highest TP was obtained at run 3, at the same temperature and extraction time as at run 6 but using 60% of ethanol. At the run 6, the TP value was 6.5480 g GAE/100 g, which indicates that the differences between those values are minimal. The same happens when it comes to the lowest values of Y and TP. The lowest value of Y was obtained at run 19, at the temperature of 130 °C and extraction time of 10 min using the 60% of ethanol. At the run 19 the TP was 4.1336 g GAE/100 g, while the minimal TP (4.0400 g GAE/100 g) was obtained at same conditions but using 30% of ethanol (run 4). Thus, there is a very small difference between TP values using 30 and 60% of ethanol at the same temperature and extraction time, where, according to *p* values, the significance of linear term of ethanol concentration on TP could be neglected (Table S1). Two other linear terms exhibited high significant effect (*p* < 0.01) on Y and TP. In comparison with TP, differences between Y values obtained at the temperature of 170 °C and extraction time of 30 min using 30 and 60% of ethanol were slightly higher (Table 2), which could be explained by the extraction of undesirable compounds due to their polarity and affinity to solvent. This effect has been previously observed in the study by Herrero et al. [21], where the TP of rosemary extracts kept increasing with increasing of extraction temperature and where the differences between Y and antioxidant activity of water and ethanol extracts at temperatures above 150 °C were minimal. The biggest differences between water and ethanol as solvents were more noticeable at lower temperatures, where water extracts showed significantly higher Y and antioxidant activity [21].

**Figure 2:**
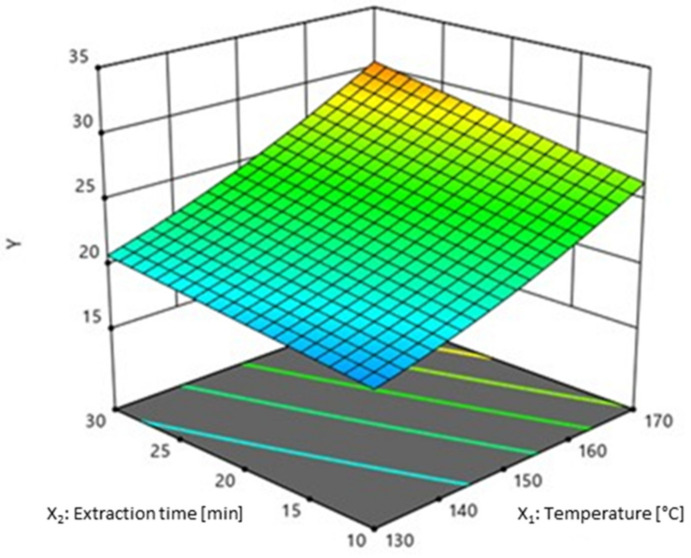
Surface plot of extraction time and temperature effect on Y.

The interaction between temperature and extraction time exhibited a significant effect, while the interaction between temperature and concentration of ethanol exhibited moderate significance (0.01 < *p* < 0.01) on Y. The surface plot in Figure 2 shows the effects of extraction time and temperature on Y. It demonstrates that the maximized Y value is not in the range of experimental data, but it must be taken into account that by increasing the temperature and prolonging the extraction time, concomitant compounds could be extracted, while also resulting in an unjustified energy consumption. The impact of temperature and type of solvent on Y was already investigated for different plant materials by Miron et al. [4], who compared Y and bioactivity of PLE extracts of three native Romanian plants, oregano (*O. vulgare*), tarragon (*A. dracunculus*) and wild thyme (*T. serpyllum*). They concluded that higher temperatures bring higher extraction yields, which is directly related to the decrease of solvent viscosity, which further affects the easier penetration into the matrix and increased mass transfer.

**Figure 3:**
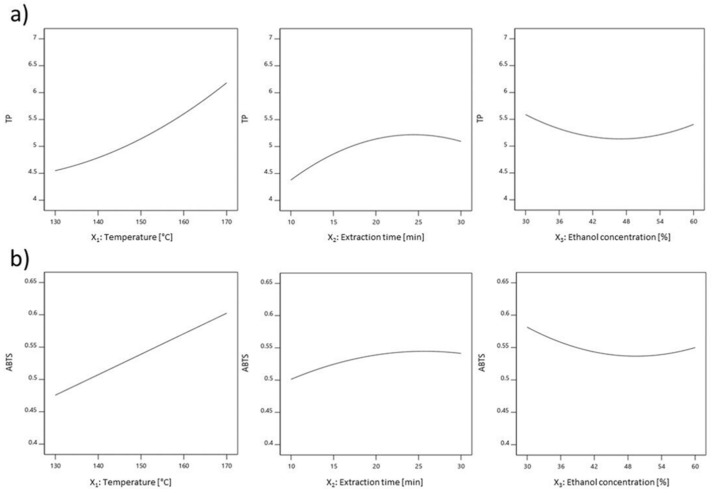
Effects of temperature, extraction time and ethanol concentration on (**a**) TP and (**b**) ABTS.

The quadratic term of temperature significantly influenced Y (Table S1). As the temperature increases, the Y value increases as well (Figure S5a), which is why it is necessary to set the temperature value as high as possible without degradation of thermolabile compounds, as well as to take into account the rationalization of energy consumption during the production process. In contrast to temperature, the quadratic term of extraction time exhibited moderately significant effect on TP, and it was in the range of experimental data, whose maximum value was obtained at extraction time of 24.45 min (Figure 3a). Quadratic term of ethanol concentration exhibited moderately significant effect on Y (Table S1). Effects of temperature, extraction time and ethanol concentration on Y, TP, DPPH, FRAP and ABTS were presented in the Figure 3 and Figure S5.

### 2.4. Antioxidant Activity of T. serpyllum Extracts

Results presented by Đukić et al. [12] showed that antioxidant activity of *T. serpyllum* extracts obtained by PLE proved to be promising, where the lowest value of IC_50_ (22.73 mg/mL) was obtained by this extraction technique. During this study, results attained by PLE were also compared with those obtained by conventional solid-liquid (IC_50_ = 36.83 mg/mL), UAE (IC_50_ = 35.47 mg/mL) and microwave-assisted extraction (MAE) (IC_50_ = 29.60 mg/mL) [12]. In comparison with our study, Đukić et al. [12] performed PLE at a pressure of 40 bar and at 140 °C in a duration of 30 min using double distilled water as a solvent. Miron et al. [4] also used PLE for polyphenols recovery from *T. serpyllum* and came to the conclusion that the highest antioxidant activity of extracts was obtained at 100 °C using mixtures of water and ethanol (25:75, 50:50 and 75:25) and at 200 °C using pure water as a solvent.

Antioxidant activity of *T. serpyllum* extracts, that was obtained by DPPH, FRAP and ABTS assays, was in the range between 0.2431 and 0.2914 mM TE/g, 0.6726 and 0.9357 mM Fe^2+^/g and 0.4362 and 0.6482 mM TE/g, respectively (Table 2). According to the results, it could be concluded that there are certain similarities when comparing them. The difference between run 3 and run 6, on which the highest values of antioxidant activity were obtained, is only the solvent, where 60% at run 3 and 30% of ethanol at run 6 was used, while the temperature was fixed at 170 °C and extraction time at 30 min. That has also been confirmed by *p* values (Table S1), where the linear term of ethanol concentration exhibited insignificant effect (*p* >> 0.05) on all three responses. The possible explanation is correlated to behavior of solvent at subcritical level, where it acquires such properties that provide it with extreme selectivity in terms of extracting the polar compounds and leading to a higher TP [18].

Similar to Y and TP, the lowest DPPH, FRAP and ABTS values were obtained at run 19, at 130 °C for 10 min using 60% of ethanol, suggesting that polyphenols are most responsible for the antioxidant activity of the obtained extracts. The same observation was concluded in other studies as well [5,6].

According to *p* values presented in the Table S1, the linear term of temperature exhibited high significance on DPPH, FRAP and ABTS. This case where the temperature was found to be the most dominant factor was in agreement with the finding of Vergara-Salinas et al. [29], who investigated the effects of temperature and extraction time on the pressurized hot water extraction of deodorized thyme (*Thymus vulgaris* L.), where the TP, DPPH and FRAP were used as output parameters. Linear term of extraction time exhibited high significance only on DPPH, while the significance on FRAP was moderate. The significance of all other linear, interaction and quadratic terms on DPPH, FRAP and ABTS could be neglected. In order to appropriately set upper values for temperature and extraction time, the potential reduction of the total polyphenols yield needs to be taken into account due to thermolability of the polyphenolic compounds, as well as possible enzymatic degradation, oxidation or polymerization [29].

### 2.5. Process Optimization and Experimental Verification

The process optimization resulted in choosing the best combination of input parameters in order to obtain the extract that possesses maximized Y and TP, as well as maximized antioxidant activity simultaneously determined in three model systems. Optimized PLE conditions were the temperature of 170 °C, extraction time of 30 min and 30% of ethanol as a solvent with desirability function of 0.913 (Table 5). The reason for limiting the temperature at 170 °C could be justified by a high risk of degradation of polyphenolic compounds at elevated temperatures, as well as extraction of undesirable compounds, which can greatly affect the downstream of the process [21].

Under optimal PLE conditions TP, DPPH and ABTS values were slightly higher in comparison with predicted ones (Table 5). Y was almost the same, but FRAP value was slightly lower than the one that was predicted. According to very good correlation between predicted and experimental values, it could be concluded that the validation of the optimization process was successfully done, and polynomial equations could be used for point prediction within investigated experimental domain.

### 2.6. Polyphenols Profile

A total of 29 different polyphenolic compounds were identified per extracts, which were obtained by PLE on the central point (Sample PLE-CP) and under the optimal conditions (Sample PLE-OPT) (Table 6).

In PLE extracts were identified gallic, vanillic, protocatechuic, *p*-coumaric, 3-*p*-coumaroylquinic, 4-*p*-coumaroylquinic, coumaric acid hexoside isomer-1, coumaric acid hexoside isomer-2, coumaric acid hexoside isomer-3 and caffeic acid as phenolic acids. Besides *p*-coumaric acid and caffeic acid and its derivatives, Boros et al. [30] in *T. serpyllum* also identified chlorogenic and ferulic acid. Furthermore, Fecka and Turek [31] detected luteolin, luteolin-7-*O*-rutinoside, luteolin-7-*O*-β-glucuronide, eriodictyol, eriocitrin, caffeic, lithospermic and rosmarinic acid in the previously obtained by UAE *T. serpyllum* methanolic extracts. In comparison with aforementioned studies, Jovanović et al. [5] identified phenolic acids in *T. serpyllum* ethanol extracts obtained by UAE, where the main one was rosmarinic acid, followed by salvianolic acid K isomer and salvianolic acid I. Additionally, authors identified 4 flavonoids (6,8-di-*C*-glucosylapigenin, 6-hydroxyluteolin-7-*O*-glucoside, luteolin-7-*O*-glucuronide and apigenin-glucuronide) as well [5].

One of the flavonoids identified in Sample PLE-CP and Sample PLE-OPT was quercetin, which was identified in a form of a hexoside isomer and a glucuronide, as well as in a form of 3-*O*-glycosides with galactose, glucose and rutinose as the carbohydrate compounds. In comparison with quercetin, kaempferol was identified in a free form and in the form of 3-rutinoside, 3-galactoside and 3-glucoside, while isorhamnetin was identified in the form of 3-*O*-galactoside only. The next flavonoid subgroup refers to flavan-3-ols, where (+)-catechin was identified in a free form only, while (−)-epicatechin was identified in a free form and in the form of 3-*O*-gallate. Naringenin, which was identified in a free form and in a form of 7-*O*-glucoside, and eriodictyol, that was also identified in a free form only, represented subgroup of flavanones. The only flavone and isoflavone identified were luteolin and biochanin A, respectively, while the only coumarin identified was dihydroxycoumarin. Luteolin in a free form and in a form of 7-*O*-glycoside, 7-*O*-β-D-glucuronide and 7-*O*-β-D-rutinoside was also identified in *T. serpyllum* by Milevskaya et al. [32]. In addition, authors identified phenolic acids (quinic, 3,4-dihydroxyphenyllactic, protocatechuic, caffeic, 3-*O*-caffeoylquinic, 4-*O*-caffeoyl- quinic, 5-*O*-caffeoylquinic, carnosic, 3,5-dicaffeoylquinic and rosmarinic acid), as well as other polyphenolic compounds like rutin, protocatechuic aldehyde, apigenin, apigenin-7-glucuronide, carnosol and methyl carnosate [32].

The only identified compound belonging to the tannin group was monogalloyl-glucose, which together with (+)-catechin and (−)-epicatechin were identified only in the Sample PLE-CP, at 150 °C for 20 min using 45% of ethanol as a solvent. However, the stilbenoid *trans*-piceatannol together with naringenin and (−)-epicatechin-3-*O*-gallate were identified only in the Sample PLE-OPT obtained due to the extraction at optimal PLE conditions, at temperature of 170 °C, extraction time of 30 min using 30% of ethanol as a solvent.

In addition, Boros et al. [30] identified polyphenols from two *T. serpyllum* samples, which were planted at different localities and in different year. Both samples contained flavanones (naringenin, eriodictyol and dihydroquercetin), flavones (apigenin), flavonols (quercetin and rutin) and flavan-3-ol catechin. Epicatechin and hesperetin were not identified in plant grown in Hungary, while apigenin-7-glucoside was not identified in plant grown in Romania. Based on the identified phenolic compounds from *T. serpyllum* grown in different places and at different times, they came to the conclusion that their polyphenols profiles of examined plants differ precisely because of the aforementioned factors [30]. It could be concluded that polyphenols profile of *T. serpyllum* is very variable depending on the climate and geographical origin, as well as on the choice of extraction technique and extraction parameters.

## 3. Materials and Methods

### 3.1. Sample

The herbal dust fraction of *T. serpyllum* L. was provided by the domestic filter-tea factory Macval D.O.O. (Novi Sad, Serbia). Dried material was stored in paper bags in a dry place at room temperature prior to extractions.

### 3.2. Chemicals

Folin-Ciocalteu reagent, (±)-6-hydroxy-2,5,7,8-tetramethylchromane-2-carboxylic acid (Trolox), gallic acid, 2,2-diphenyl-1-picrylhydrazyl (DPPH) and 2,4,6-tris(2-pyridyl)-s-triazine (≥99.0%) were supplied from Sigma-Aldrich (Steinheim, Germany). 2,2′-Azino-bis(3-ethylbenzothiazoline-6-sulfonic acid) diammoniumsalt (98%) was purchased from J&K, Scientific Ltd. (Beijing, China). Additionally, sodium carbonate anhydrous and ferric chloride hexahydrate were supplied from Centrohem (Stara Pazova, Serbia), while acetic acid (99.8%) and potassium peroxydisulfate were purchased from Lach-Ner (Neratovice, Czech Republic). Sodium acetate anhydrous was purchased from Kemika (Zagreb, Croatia). Ultra-pure water was obtained from a Milli-Q Plus system (EMD Millipore, Billerica, MA, USA). All other chemicals used were of analytical reagent grade.

### 3.3. PLE

PLE was conducted using an accelerated solvent extractor (ASE 350, Dionex, Sunnyvale, CA, USA). In each experimental run, 4 g of *T. serpyllum* dust and 1 g of diatomaceous earth as a desiccant were mixed and placed into a 22 mL stainless steel extraction cell. The PLE experiments were conducted at fixed pressure (1500 psi) and at fixed purge time with N_2_ (90 s). Concentrations of aqueous ethanol used as solvent, temperature, static extraction time, cell flush volume and number of cycles were varied in screening experiments, while ethanol concentration, temperature and extraction time were evaluated in RSM study. Obtained extracts were diluted by solvent in order to adjust solid/liquid ratio to 1:20, *w*/*v*. Obtained samples were then collected into plastic vials and stored at 4 °C prior to analysis.

### 3.4. Determination of Y and TP

Y in extracts was determined by the vacuum vaporization process of 10 mL of crude extract and further drying in the oven at 105 °C until constant mass achieved. Results were presented as a mass of total extractable solids per 100 g of dry plant material (%; *w/w*). TP in all extracts was determined by spectrophotometric method using the Folin-Ciocalteu assay [33]. Absorbances were recorded at 750 nm using a spectrophotometer (model 6300, Jenway, Stone, UK). All experiments were performed in triplicate and mean values of the TP of obtained extracts were presented as grams of gallic acid equivalents (GAE) per 100 g of sample dry weight (g GAE/100 g).

### 3.5. Antioxidant Activity of Extracts

Antioxidant activity was determined by DPPH, FRAP and ABTS assays. Antioxidant activity towards DPPH radicals was determined by spectrophotometric method [34]. Obtained extract (100 μL) was added to 2900 μL of DPPH solution, which was previously prepared in concentration of 26 mg/L of methanol. After 1 h, the absorbances were recorded at wavelength of 517 nm. Mean values of the antioxidant potential were presented as mM of Trolox equivalents (TE) per g of sample dry weight (mM TE/g).

The reducing power of the extracts was determined by the ferric ion reducing antioxidant power (FRAP) assay [35]. FRAP reagent was prepared by mixing 10 mM/L 2,4,6-tripyridil-s-triazine in 40 mM/L HCl, 20 mM/L FeCl_3_, and acetate buffer, pH 3.6, in ratio of 1:1:10, respectively. Obtained extract (100 µL) was added to 2900 µL of FRAP reagent. After incubation in the dark at 37 °C for 10 min, the absorbances were recorded at wavelength of 593 nm. Mean values of reducing power were presented as mM of Fe^2+^ per g of sample (mM Fe^2+^/g).

The scavenging capacity towards ABTS^+^ radical of PLE extracts was determined by spectrophotometric method [36]. ABTS stock solution was freshly prepared from a mixture (1:1, *v*/*v*) of 2.45 mM potassium persulfate aqueous solution and 7 mM ABTS (2,2′-azino-bis-(-3-ethylbenzothiazoline-6-sulfonic acid) diammonium salt) aqueous solution and left in the dark at room temperature for 16 h. A stock solution was diluted using acetate buffer (pH 3.6) to an absorbance of 0.70 (±0.02) at wavelength of 734 nm. Obtained extract (100 µL) was added to 2900 µL of ABTS reagent and stored in the dark at room temperature for 5 h. Mean values of antioxidant activity towards ABTS^+^ radical were presented as mM of Trolox equivalents (TE) per g of sample dry weight (mM TE/g). All experiments were performed in triplicate.

### 3.6. Q Exactive Hybrid Quadrupole-Orbitrap LC-MS/MS Analysis

In order to test free, ester-linked and glycoside-linked phenolic compounds in samples obtained by PLE, the Q Exactive LC-MS/MS—Orbitrap (Thermo Scientific, Hemel Hempstead, UK) was employed. Chromatographic separation of compounds was achieved on a Poroshell 120 EC-C18 column (3.0 × 100 mm, 2.7 µm, Agilent, Santa Clara, CA, USA) setting gradient flow at 0.6 mL/min (mobile phase A: 0.1% formic acid-water, mobile phase B: methanol; 0–5 min, mobile phase B concentration changed as 0–9% B; 5–9 min, 9–2% B; 16–35 min, 2–18% B; 35–50 min, 18–20% B; 50–65 min, 20–30% B and 65–80 min, 30% B). The injection volume was 10 µL. Q Exactive hybrid quadrupole-Orbitrap mass spectrometer equipped with an ESI source working in both negative and positive ionization mode was used for accurate mass measurements. Following parameters were set: ion spray voltage, 2.8 kV; capillary temperature, 300 °C; capillary voltage, 35 V and tube lens voltage, 95 V; sheath gas, 19 (arbitrary units); auxiliary gas, 7 (arbitrary units). Mass spectra were recorded covering the m/z range of 55–1000 Da. Default values were used for most other acquisition parameters (automatic gain control (AGC) target 3 × 10^6^ ions). The data processing was achieved using XCalibur 2.2 software (Thermo Fisher Scientific, Waltham, MA, USA). An external calibration for mass accuracy was performed before the analysis. The same method was described in detail by Pavlić et al. [37].

### 3.7. Design of Experiments and Statistical Methods

The first step was screening of the independent variables in order to define the most influential parameters and their domain on already determined responses (Y, TP, DPPH) using 2^5-1^ fractional factorial design. Design of experiments consisted of 16 runs, where the temperature (80 and 150 °C), extraction time (5 and 20 min), ethanol concentration (40 and 80%) and rinse volume (50 and 100%) were used as numerical, and number of cycles (1 and 3) as categorical independent variables. In order to determine the impact of PLE parameters on Y and TP, as well as antioxidant activity (DPPH), the linear model given by Equation (1) was used:

(1) $$Y=\beta_{0}+\overset{5}{\underset{i=1}{\sum }}\beta_{i}X_{i}+\overset{4}{\underset{i=1}{\sum }}\overset{5}{\underset{j=i+1}{\sum }}\beta_{\mathrm{ij}}X_{i}X_{j}$$

where Y represents the response variable, β_0_ the intercept, β_i_ the linear regression coefficient, β_ij_ the regression coefficients for cross-product terms and X_i_ and X_j_ the independent variables affecting the response.

After the screening, three of the five most influential parameters were selected, which were further used in face-centered CCD with RSM. The impact of temperature (130, 150 and 170 °C), extraction time (10, 20 and 30 min) and ethanol concentration (30, 45 and 60%) were used as independent variables. Optimal extraction conditions were determined considering Y and TP, as well as antioxidant activity parameters obtained by DPPH, ABTS and FRAP assays, while selection of optimal conditions were based on desirability function (*D*) [38]. For multiple linear regression analysis Design-Expert v.11 software (Stat-Ease, Minneapolis, MN, USA) was used and results were fitted to a second-order polynomial model (Equation (2)):

(2) $$Y={\;\beta }_{0}+\overset{3}{\underset{i=1}{\sum }}\beta_{i}X_{i}+\overset{3}{\underset{i=1}{\sum }}\beta_{\mathrm{ii}}X_{i}^{2}+\overset{2}{\underset{i=1}{\sum }}\overset{3}{\underset{j=i+1}{\sum }}\beta_{\mathrm{ij}}X_{i}X_{j}$$

where Y represents the response variable, X_i_ and X_j_ are the independent variables affecting the response, and β_0_, β_i_, β_ii_, and β_ij_ are the regression coefficients for intercept, linear, quadratic and cross-product terms. The goodness of fit was determined by ANOVA, while model adequacy was evaluated by the *R*^2^, CV and *p*-values for the model and lack of fit. In order to verify obtained empirical models, validation was performed by using the extracts prepared at optimized PLE conditions.

## 4. Conclusions

Through the first screening phase of PLE parameters, three most influential PLE parameters (temperature, extraction time and ethanol concentration) were selected for the second phase of research (RSM study). The applied quadratic model provided adequate mathematical description of PLE of the investigated responses, Y, TP and antioxidant activity parameters obtained by DPPH, FRAP and ABTS assays. Concerning all of investigated responses, extraction temperature had the most dominant positive influence, followed by positive influence of extraction time and negative effect of ethanol concentration. Increasing the temperature above 170 °C and prolonging the extraction time above 30 min led to an increase in probability of extraction of concomitant compounds and unjustified energy consumption, which is why it is necessary to satisfy techno-economical aspect and streamline the production by limiting aforementioned parameters. Maximized Y, TP and antioxidant activity of *T. serpyllum* extracts were obtained at the temperature of 170 °C, extraction time of 30 min and 30% of ethanol as a solvent. It could be concluded that PLE technique in combination with RSM can be successfully applied for the extraction of biologically active compounds from *T. serpyllum* by-product, which represents the source of natural antioxidants with great potential for further use in various forms within different branches of industry.

## Figures and Tables

**Table 1:** 2^5-1^ fractional factorial design with coded and actual values of input parameters and experimentally observed values of investigated responses.

Run	Input Parameters										Responses		
	X_1_: Temperature [°C]		X_2_: Extraction Time [min]		X_3_: Ethanol Concentration [%]		X_4_: Cell Flush Volume [%]		X_5_: Cycle		Y [%]	TP [g GAE/100 g]	DPPH [mM TE/g]
1	−1	80	−1	5	1	80	1	100	E2	3	10.97	3.3438	0.1382
2	−1	80	−1	5	1	80	−1	50	E1	1	8.30	2.1234	0.0700
3	1	150	1	20	−1	40	−1	50	E2	3	28.87	5.9828	0.2224
4	1	150	−1	5	1	80	1	100	E1	1	16.08	4.1972	0.1651
5	−1	80	−1	5	−1	40	1	100	E1	1	13.98	3.7593	0.1503
6	−1	80	1	20	1	80	1	100	E1	1	11.21	2.8085	0.1257
7	1	150	−1	5	1	80	−1	50	E2	3	17.38	4.3582	0.1802
8	1	150	1	20	1	80	1	100	E2	3	20.60	4.7363	0.1838
9	−1	80	1	20	−1	40	1	100	E2	3	17.60	4.5528	0.1785
10	−1	80	1	20	−1	40	−1	50	E1	1	14.03	4.0213	0.1656
11	1	150	1	20	−1	40	1	100	E1	1	23.74	5.4550	0.2105
12	−1	80	−1	5	−1	40	−1	50	E2	3	15.11	3.8978	0.1358
13	1	150	−1	5	−1	40	1	100	E2	3	22.65	5.3127	0.2059
14	1	150	1	20	1	80	−1	50	E1	1	18.08	4.6315	0.1875
15	−1	80	1	20	1	80	−1	50	E2	3	12.19	2.9095	0.1323
16	1	150	−1	5	−1	40	−1	50	E1	1	20.81	4.6689	0.1936

**Table 2:** Face-centered CCD with three levels of PLE parameters and experimentally observed values of investigated responses.

Run	Input Parameters						Responses				
	X_1_: Temperature [°C]		X_2_: Extraction Time [min]		X_3_: Ethanol Concentration [%]		Y [%]	TP [g GAE/100 g]	DPPH [mM TE/g]	FRAP [mM Fe^2+^/g]	ABTS [mM TE/g]
1	1	170	−1	10	−1	30	28.21	6.2186	0.2824	0.8429	0.6011
2	0	150	0	20	0	45	23.46	5.4063	0.2733	0.7969	0.6011
3	1	170	1	30	1	60	27.33	6.6454	0.2914	0.9357	0.6391
4	−1	130	−1	10	−1	30	19.80	4.0400	0.2506	0.7156	0.5078
5	0	150	−1	10	0	45	21.68	4.5379	0.2605	0.7205	0.5186
6	1	170	1	30	−1	30	32.54	6.5480	0.2903	0.9327	0.6482
7	1	170	0	20	0	45	28.26	6.0240	0.2818	0.7975	0.6228
8	0	150	0	20	1	60	21.10	5.4924	0.2767	0.7969	0.5540
9	−1	130	1	30	1	60	18.56	4.7512	0.2561	0.6726	0.5005
10	0	150	0	20	−1	30	24.31	5.3090	0.2710	0.7532	0.5838
11	1	170	−1	10	1	60	23.61	5.2940	0.2773	0.8296	0.5494
12	−1	130	0	20	0	45	19.76	4.5154	0.2603	0.7532	0.4860
13	0	150	0	20	0	45	23.22	5.5935	0.2720	0.7623	0.5476
14	0	150	0	20	0	45	23.34	5.0657	0.2824	0.7872	0.5340
15	0	150	0	20	0	45	23.46	5.1967	0.2739	0.7841	0.5404
16	0	150	0	20	0	45	22.28	4.8972	0.2567	0.7726	0.4896
17	0	150	1	30	0	45	25.41	4.7438	0.2810	0.8163	0.5304
18	0	150	0	20	0	45	23.68	5.0657	0.2769	0.7865	0.4842
19	−1	130	−1	10	1	60	16.17	4.1336	0.2431	0.6962	0.4362
20	−1	130	1	30	−1	30	21.64	5.1218	0.2693	0.8029	0.4960

**Table 3:** ANOVA table.

Response	Source	Sum of Squares	df	Mean Square	*F*-Value	*p*-Value
Y	Model	263.27	7	37.6100	212.94	<0.0001
	Residual	2.1200	12	0.1766		
	Lack of Fit	0.8917	7	0.1274	0.5188	0.7922
	Pure Error	1.2300	5	0.2456		
	Cor Total	265.3900	19			
	*R*^2^ = 0.992					
	CV = 1.8%					
TP	Model	8.86	7	1.2700	15.46	< 0.0001
	Residual	0.9826	12	0.0819		
	Lack of Fit	0.6576	7	0.0939	1.4400	0.3542
	Pure Error	0.3251	5	0.0650		
	Cor Total	9.8400	19			
	*R*^2^ = 0.900					
	CV = 5.47%					
DPPH	Model	0.0027	7	0.0004	9.35	0.0005
	Residual	0.0005	12	0.0000		
	Lack of Fit	0.0001	7	0.0000	0.2527	0.9496
	Pure Error	0.0004	5	0.0001		
	Cor Total	0.0032	19			
	*R*^2^ = 0.845					
	CV = 2.38%					
FRAP	Model	0.071	9	0.0079	6.24	0.0042
	Residual	0.0126	10	0.0013		
	Lack of Fit	0.0119	5	0.0024	15.9000	0.0043
	Pure Error	0.0007	5	0.0001		
	Cor Total	0.0836	19			
	*R*^2^ = 0.849					
	CV = 4.51%					
ABTS	Model	0.0517	7	0.0074	8.34	0.0008
	Residual	0.0106	12	0.0009		
	Lack of Fit	0.0015	7	0.0002	0.1142	0.9936
	Pure Error	0.0092	5	0.0018		
	Cor Total	0.0623	19			
	*R*^2^ = 0.829					
	CV = 5.47%					

**Table 4:** Reduced predictive model equations for target responses.

Response	Model Equation
Y	$Y=23.31+4.4X_{1}+1.6X_{2}-1.97X_{3}+0.4782X_{1\;}X_{2}-0.3891X_{1}X_{3}+0.736X_{1}^{2}-0.5672X_{3}^{2}$
TP	$\mathrm{TP}=5.14+0.8168X_{1}+0.3586X_{2}-0.0921X_{3}-0.0688X_{1}X_{3}+0.226X_{1}^{2}-0.4029X_{2}^{2}+0.357X_{3}^{2}$
DPPH	$\mathrm{DPPH}=0.2728+0.0144X_{1}+0.0074X_{2}-0.0019X_{3}-0.0012X_{1\;}X_{2}+0.0021X_{1}X_{3}-0.0012X_{1}^{2}-0.0016X_{2}^{2}$
FRAP	$\mathrm{FRAP}=0.7746+0.0698X_{1}+0.0355X_{2}-0.0116X_{3}+0.0165X_{1\;}X_{2}+0.0174X_{1}X_{3}-0.0118X_{2}X_{3}+0.0112X_{1}^{2}+0.0042X_{2}^{2}+0.0109X_{3}^{2}$
ABTS	$\mathrm{ABTS}=0.539+0.0634X_{1}+0.0201X_{2}-0.0158X_{3}+0.0105X_{1\;}X_{2}+0.0148X_{2}X_{3}-0.0177X_{2}^{2}+0.0267X_{3}^{2}$

X_1_—Temperature; X_2_—Extraction time; X_3_—Ethanol concentration.

**Table 5:** Predicted and experimental values of investigated responses obtained at optimal PLE conditions.

Input and Output Parameters	Goal	Lower Limit	Upper Limit	Predicted Values	Experimental Values
				Optimal Conditions	
Temperature [°C]	is in range	130	170	170	
Extraction time [min]	is in range	10	30	30	
Ethanol concentration [%]	is in range	30	60	30	
Y [%]	maximize	16.17	32.54	32.32	32.14
TP [g GAE/100 g]	maximize	4.0400	6.6454	6.6560 ± 0.4595	6.7464 ± 0.1860
DPPH [mM TE/g]	maximize	0.2431	0.2914	0.2900 ± 0.0115	0.3173 ± 0.0035
FRAP [mM Fe^2+^/g]	maximize	0.6726	0.9357	0.9290 ± 0.0706	0.8587 ± 0.0032
ABTS [mM TE/g]	maximize	0.4362	0.6482	0.6430 ± 0.0528	0.6943 ± 0.0204

**Table 6:** Polyphenols profile of extracts obtained by PLE on the central point (Sample PLE-CP) and under the optimal conditions (Sample PLE-OPT).

Retention Time [min]	Compound	Sample PLE-CP	Sample PLE-OPT
		Measured Mass [*m*/*z*]/Error [mDa]	
12.67	Monogalloyl-glucose	331.07/0.39	ND
14.14	Gallic acid	169.01/−0.17	169.01/0.28
16.15	Vanillic acid	167.03/−0.41	167.03/−0.32
19.28	Protocatechuic acid	153.02/−0.08	153.02/0.62
34.67	*trans*-Piceatannol	ND	243.07/0.33
37.61	3-*p*-Coumaroylquinic acid	337.09/0.65	337.09/0.38
37.61	4-*p*-Coumaroylquinic acid	337.09/0.65	337.09/0.38
39.33	(+)-Catechin	289.07/0.38	ND
39.33	(−)-Epicatechin	289.07/0.38	ND
39.53	Coumaric acid hexoside isomer-1	325.09/0.02	325.09/0.49
39.53	Coumaric acid hexoside isomer-2	325.09/0.02	325.09/0.49
39.53	Coumaric acid hexoside isomer-3	325.09/0.02	325.09/0.49
39.53	*p*-Coumaric acid	163.04/0.18	163.04/0.28
43.32	Dihydroxycoumarin	177.02/−0.33	177.02/0.36
44.46	Caffeic acid	179.03/−0.21	179.03/−0.29
66.28	Quercetin hexoside isomer-1	463.09/0.32	463.09/0.72
66.28	Quercetin hexoside isomer-2	463.09/0.32	463.09/0.72
66.28	Quercetin-3-*O*-galactoside	463.09/0.32	463.09/0.72
66.28	Quercetin-3-*O*-glucoside	463.09/0.32	463.09/0.72
66.61	Kaempferol-3-rutinoside	593.15/−0.52	593.15/0.71
72.90	Naringenin-7-*O*-glucoside	433.11/0.20	433.11/0.27
76.06	Quercetin-3-*O*-rutinoside	609.15/0.01	609.15/0.81
76.37	Quercetin glucuronide	477.07/1.69	477.07/1.69
76.45	Kaempferol-3-galactoside	447.09/−0.25	447.09/0.10
76.45	Kaempferol-3-glucoside	447.09/−0.25	447.09/0.10
76.52	Isorhamnetin-3-*O*-galactoside	477.10/0.07	477.10/−0.76
76.83	Eriodictyol	287.06/0.89	287.06/0.68
77.27	Naringenin	ND	271.06/0.86
77.54	(−)-Epicatechin-3-*O*-gallate	ND	441.08/3.97
78.10	Kaempferol	285.04/0.02	285.04/0.13
78.10	Luteolin	285.04/0.02	285.04/0.13
78.68	Biochanin A	283.06/−0.15	283.06/0.50

ND = Not Detected.

## Data Availability

Data is contained within this article and Supplementary Material.

## Supplementary Materials

The following are available online. Figure S1: Pareto chart exhibiting effects of five PLE variables on TP, Figure S2: Influence of temperature, extraction time, ethanol concentration, rinse volume and number of cycles on Y, Figure S3: Influence of temperature, extraction time, ethanol concentration, rinse volume and number of cycles on TP, Figure S4: Influence of temperature, extraction time, ethanol concentration, rinse volume and number of cycles on DPPH, Figure S5: Effects of temperature, extraction time and ethanol concentration on (a) Y, (b) DPPH and (c) FRAP, Table S1: Significance of linear, cross product and quadratic terms on Y, TP, DPPH, FRAP and ABTS.

## Sample Availability

Samples of the compounds are not available from authors.
