# Supplementary material for: Pressurized-Liquid Extraction as an Efficient Method for Valorization of Thymus serpyllum Herbal Dust towards Sustainable Production of Antioxidants
Source: Molecules. 2021 Apr 27;26(9):2548. doi: 10.3390/molecules26092548 (PMC8123860; doi:10.3390/molecules26092548)
Supplement: Supplementary file 1 [file molecules-26-02548-s001.zip › molecules-1183466-supplementary.pdf]

Supplementary Material

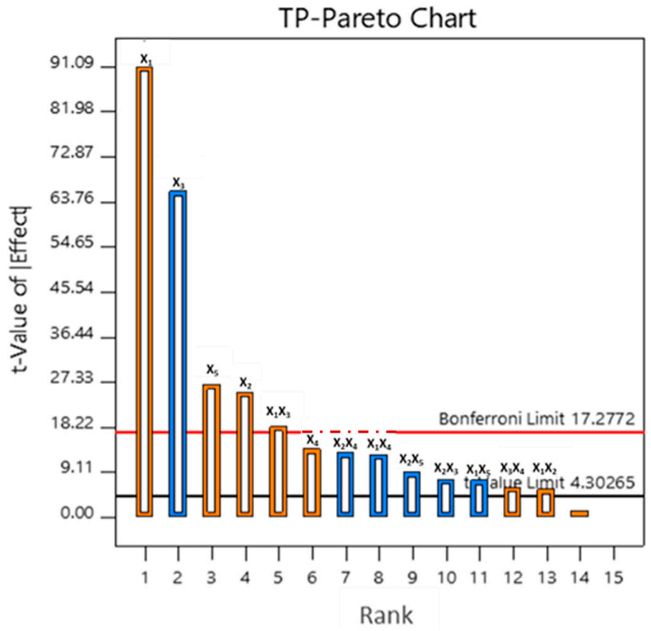

Figure S1. Pareto chart exhibiting effects of five PLE variables on TP

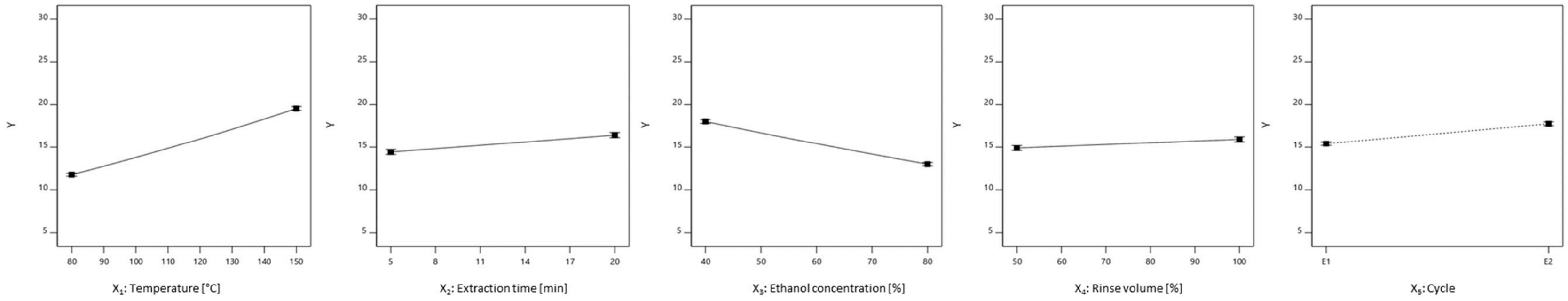

Figure S2. Influence of temperature, extraction time, ethanol concentration, rinse volume and number of cycles on Y

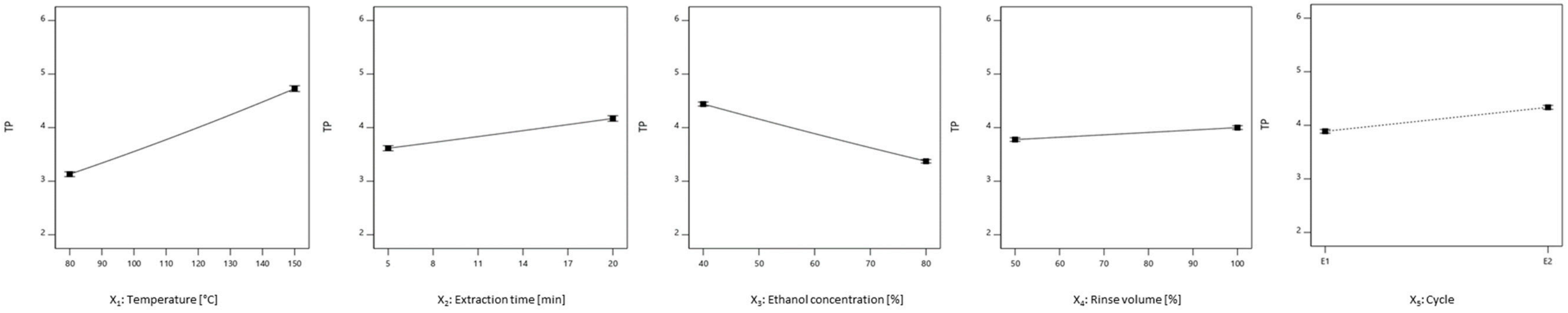

Figure S3. Influence of temperature, extraction time, ethanol concentration, rinse volume and number of cycles on TP

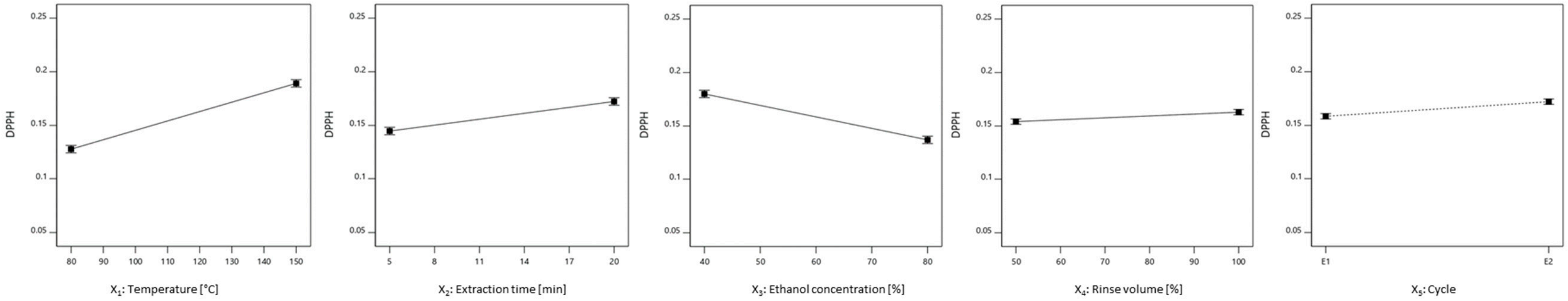

Figure S4. Influence of temperature, extraction time, ethanol concentration, rinse volume and number of cycles on DPPH

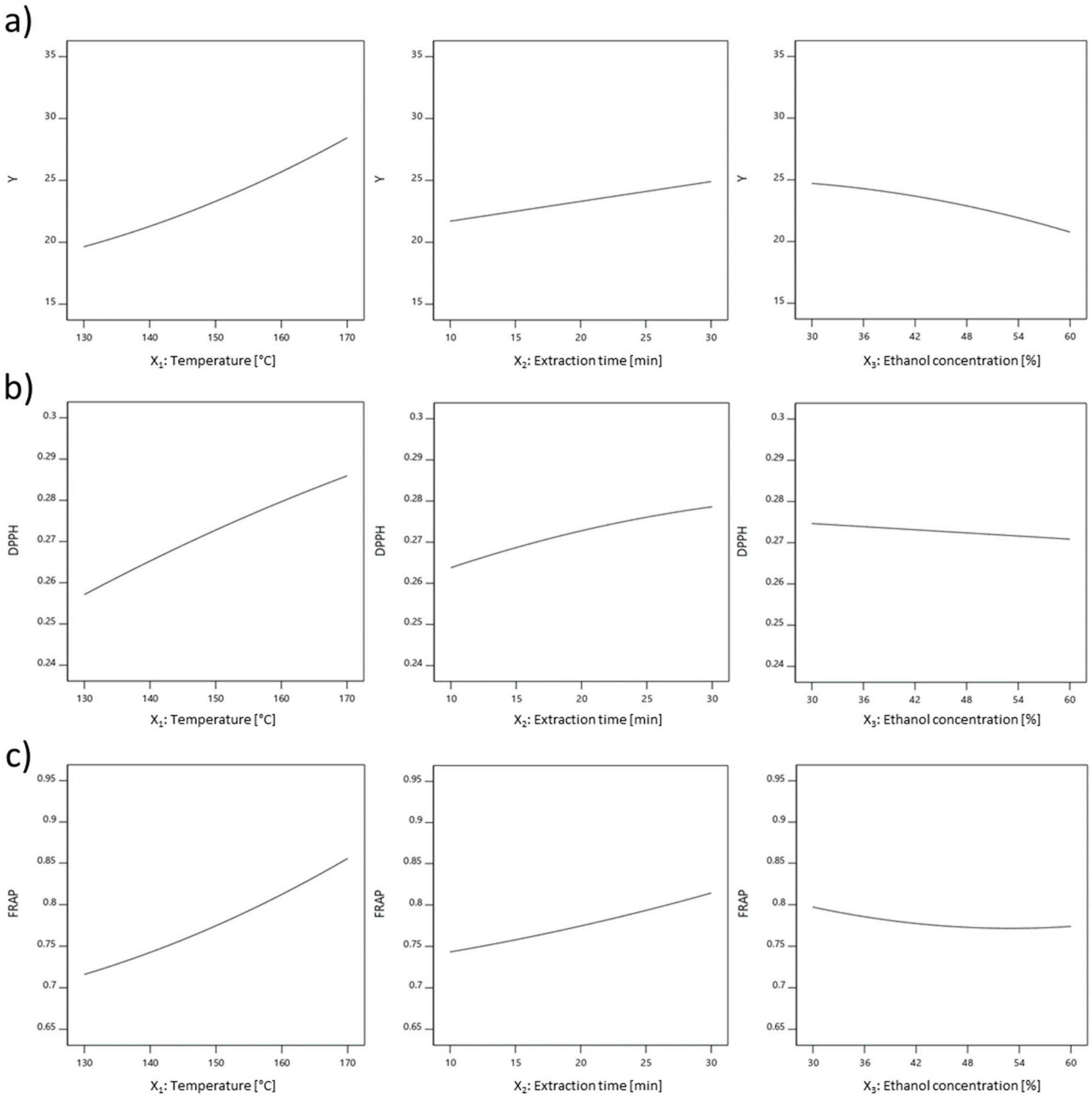

Figure S5. Effects of temperature, extraction time and ethanol concentration on **a**) Y, **b**) DPPH and **c**) FRAP

Table S1. Significance of linear, cross product and quadratic terms on Y, TP, DPPH, FRAP and ABTS.

Coefficients	Y	TP	DPPH	FRAP	ABTS
			p-value		
Linear					
X_1-Temperature	<0.0001 *	<0.0001 *	<0.0001 *	0.0001 *	<0.0001 *
X_2-Extraction time	<0.0001 *	0.0019 *	0.0035 *	0.0102 **	0.0539
X_3-Ethanol concentration	<0.0001 *	0.3289	0.3728	0.3249	0.1198
Cross product					
X_1X_2	0.0074 *	-	0.6025	0.2181	0.3366
X_1X_3	0.0224 **	0.5095	0.3822	0.1957	-
X_2X_3	-	-	-	0.3691	0.1840
Quadratic					
X_1^2	0.0086 *	0.2149	0.7356	0.6131	-
X_2^2	-	0.0377 **	0.6720	0.8480	0.3092
X_3^2	0.0326 **	0.0608	-	0.6226	0.1342

*$p < 0.01$ – significant

**$0.01 < p < 0.05$ – moderately significant
